# How Radioactive Iodine Treatment Affects the Retina

**DOI:** 10.1055/s-0043-1774419

**Published:** 2023-12-04

**Authors:** Ceren Gürez, Aynur Özen, Özgül Ekmekçioğlu

**Affiliations:** 1 Ophthalmology Clinic, Beyoglu Eye Training and Research Hospital, Health of Sciences University, Turkey; 2 Nuclear Medicine Clinic, Bagcilar Training and Research Hospital, Health of Sciences University, Turkey; 3 Nuclear Medicine Clinic, Sisli Hamidiye Etfal Training and Research Hospital, Health of Sciences University, Turkey

**Keywords:** Macula, nerve fiber layer, radioactive iodine, thyroid cancer, hyperthyroidism

## Abstract

**Objective** The aim of this study was to quantitatively assess the macular and retinal nerve fiber layer thicknesses in patients with hyperthyroidism and thyroid cancer undergoing radioactive iodine (RAI) therapy.

**Study Design** This prospective study was conducted in accordance with the principles of the Declaration of Helsinki and approved by the Bagcilar Training and Research Hospital Clinical Research Ethics Committee. Written informed consent was obtained from the patients following a detailed explanation of the study objectives and protocol. Patient selection was randomized. Patients scheduled for RAI treatment in the Nuclear Medicine Clinic were referred to the ophthalmology clinic, respectively. Patients without additional ocular pathology were included in the study.

**Methods** All patients had received RAI therapy using Iodine-131 for hyperthyroidism or thyroid cancer. A complete ophthalmological examination and measurement of macular and retinal nerve fiber layer thickness using optical coherence tomography were performed on all patients before and at the first and sixth months and in first year after RAI treatment. The results were prospectively evaluated.

**Results** The study included 80 eyes of 40 patients. The hyperthyroid group was group 1, and the thyroid cancer group was group 2. There were 25 patients in group 1 and 15 patients in group 2. The mean age was 43.76 ± 11.85 years (range: 22–65 years) in group 1 and 39.87 ± 9.13 years (range: 30–58 years) in group 2. There was no significant difference between the two groups regarding age and sex ( *p* > 0.05). In both groups, no significant difference was found in the macular thickness and retinal nerve fiber layer thicknesses values obtained in both eyes before and after the RAI treatment.

**Conclusion** As a result of our study, we observed that RAI intake did not harm the retinal layer.

## Introduction

Radioactive iodine (RAI) is a safe, effective, noninvasive, and simple ablation treatment recommended for patients diagnosed with hyperthyroidism or papillary and follicular thyroid carcinoma. 1 2 This treatment eliminates residual normal or tumoral thyroid cells and allows post-treatment screening to reveal previously unidentified or additional disease foci. 3 4 5 6 Although it is a repeatable and generally well-tolerated treatment, RAI can also accumulate in extrathyroidal tissues, such as gastric mucosa, intestine, choroid plexus, salivary glands, sweat glands, and lacrimal glands, and may be associated with complications such as transient neck pain and edema, as well as dysfunctions in the pulmonary, gastrointestinal, and hematopoietic systems, and damages to salivary glands, nasolacrimal apparatus, and gonads, along with the potential risk of secondary malignancies. 7 8 9 10 11 12 While these complications are rarely life-threatening, they can negatively impact the patient's quality of life.

There have been several studies on the effects of RAI treatment on the lacrimal gland, which are related to the accumulation of iodine in the eye. However, there is currently a lack of known studies on its effects on the retina, which could be expected to be affected due to choroidal plexus involvement. This study aimed to conduct a quantitative assessment of the macular and retinal nerve fiber layer thicknesses (RNFLT) before and after RAI treatment in patients with hyperthyroidism and thyroid cancer, utilizing optical coherence tomography (OCT).

## Patients and Methods

This prospective study adhered to the principles outlined in the Declaration of Helsinki, and the study protocol received approval from the Bioethical Commission of The Health Sciences University Bagcilar Research and Training Hospital. Patients were enrolled in the study after providing written informed consent. The patient selection process was randomized. Specifically, patients scheduled for RAI treatment in the Nuclear Medicine Clinic were referred to the ophthalmology clinic. Only patients without any additional ocular pathology were included in the study. Exclusion criteria for all patients encompassed a history of significant ocular surface disease or ocular inflammation, thyroid eye disease, diabetes mellitus, and ocular surgery within the past year.

The study comprised 80 eyes of 40 patients, consisting of 7 men and 33 women. The participants were divided into two groups: group 1, consisting of 25 patients with hyperthyroidism, and group 2, comprising 15 patients with thyroid cancer. The mean age was 43.76 ± 11.85 years (ranging from 22 to 65 years) for group 1 and 39.87 ± 9.13 years (ranging from 30 to 58 years) for group 2. Diagnosing hyperthyroidism was clinically confirmed through laboratory evaluations, thyroid scintigraphy, and RAI uptake values in all patients. For RAI therapy, all patients received Iodine-131, with group 1 receiving low-dose (up to 999 Mega Bequere—MBq) and group 2 receiving high-dose (3,700 MBq and higher) Iodine-131. The mean RAI dose in the hyperthyroid group was 603.1 MBq (ranging from 481 to 740 MBq); in the cancer group, it was 3,700 MBq (mean and total). All patients were prescribed a low iodine content diet 10 days before the therapy, and antithyroid medications were withdrawn 7 days before the therapy. 1 2 RAI treatment was administered orally to all patients. Notably, all thyroid cancer patients and five hyperthyroid patients underwent surgery for their respective diseases ( *p* = 0.0001).

In all patients, a complete ophthalmological examination and measurement of macular and RNFLT using OCT were performed before RAI treatment and at the first and sixth months and first year post-RAI treatment. The results were prospectively evaluated.

In this study, statistical analysis was conducted using the Number Cruncher Statistical System 2007 Statistical Software (Utah, United States) pocket program. The data were evaluated using descriptive statistics (mean, standard deviation), repeated variance analysis for multiple group comparisons, and the Newman–Keuls multiple comparison test for subgroup comparisons. Additionally, an independent *t* -test was employed to compare paired groups, while the chi-square and Fisher's exact tests were used to compare qualitative data. The results were evaluated at a significance level of *p* -value less than 0.05.

### Imaging

OCT (Nidek RS-3000) was utilized to obtain cross-sectional retina measurements through dilated pupils. This instrument employed partial coherence interferometry technology, resulting in optical A-scans of the retina with an axial resolution of approximately 10 µm. 13 14 15 The peripapillary RNFL thickness was measured using the “fast RNFL thickness” scan protocol, which involved 256 sampling points along a circular scan path. Three scans were conducted, and the average value was used for analyses. The “fast macular scan” protocol was employed for macular thickness measurements to measure the distance between the internal limiting membrane and the retinal pigment epithelium. The macula center and 1-, 3-, and 6-mm rings in different segments (superior, inferior, nasal, and temporal) were measured, and an average of five scans were used for analysis. A single-experience operator performed all scans.

## Results

The age and sex characteristics of both groups were similar. In Group 1, only five patients underwent surgery, while all patients in Group 2 underwent surgery ( Table 1 ).

**Table 1:** Characteristics of the two groups

		Group 1 ( *n* = 25)		Group 2 ( *n* = 15)		*p* -Value
Age(years)		43.76 ± 11.85		39.87 ± 9.13		0.282
Sex	Female	19	76.00%	14	93.33%	0.162
	Male	6	24.00%	1	6.67%	
Operation	No	20	80.00%	0	0%	0.0001 a
	Yes	5	20.00%	15	100%	

a *p* -Value < 0.05.

In both groups, no significant difference was observed in macular thickness and RNFLT values before and after RAI treatment. Since the results were nearly identical in both eyes, the right eye was used for the analysis. In Group 1, the pretreatment values were as follows: average macular center thickness was 223.84 ± 16.8 µm, average macular 6-mm ring area thickness was 252.72 ± 14.77 µm, and average RNFLT was 107.52 ± 7.47 µm. At the end of 6 months, these values were found to be 223.42 ± 13.18 µm, 250.66 ± 12.93 µm, and 105.48 ± 15.25 µm, respectively ( *p* > 0.05 for all three tests).

In Group 2, the pretreatment values were as follows: average macular center thickness was 230.53 ± 14.72 µm, average macular 6-mm ring area thickness was 250.4 ± 14.09 µm, and average RNFLT was 109.27 ± 5.19 µm. At the end of 6 months, these values were found to be 229.4 ± 16.67 µm, 249.7 ± 12.28 µm, and 109.1 ± 4.51 µm, respectively ( *p* > 0.05 for all three tests) ( Table 2 ).

**Table 2:** Comparison between beginning and end-of-treatment macular center thickness, macular 6-mm ring area thickness, and RNFLT for the two groups

	Group 1 ( *n* = 25)	Group 2 ( *n* = 15)	*p* -Value
Macular CT Pre-t	223.84 ± 16.80	230.53 ± 14.72	0.075
Macular CT 1 month	224.00 ± 14.98	228.57 ± 18.27	0.228
Macular CT 6 month	223.42 ± 13.18	229.40 ± 16.67	0.080
*p* -Value	0.815	0.272	
RNFLT Pre-t	107.52 ± 7.47	109.27 ± 5.19	0.263
RNFLT 1 month	107.80 ± 7.29	108.63 ± 5.68	0.594
RNFLT 6 month	105.48 ± 15.25	109.10 ± 4.51	0.210
*p* -Value	0.325	0.855	
Macular 6-mm T Pre-t	252.72 ± 14.77	250.40 ± 14.09	0.491
Macular 6-mm T 1 month	250.60 ± 12.91	250.27 ± 12.66	0.911
Macular 6-mm T 6 month	250.66 ± 12.93	249.70 ± 12.28	0.744
*p* -Value	0.055	0.657	

Abbreviations: Macular 6-mm T, macular 6-mm ring area thickness; Macular CT, macular center thickness; Pre-t, pretreatment; RNFLT, retinal nerve fiber layer thicknesses.

## Discussion

To our knowledge, no other study has addressed macular and retinal nerve fiber layer thicknesses in patients with hyperthyroidism and thyroid cancer undergoing RAI therapy. However, some studies focus on the adverse effects of radioiodine therapy for differentiated thyroid carcinoma and hyperthyroidism in the eye. These studies have reported side effects, particularly related to the lacrimal system. 7 16 17 Additionally, there are studies on the adverse effects of iodine brachytherapy (IBT) applied in uveal melanomas. 18 19 20 21 Furthermore, a study on radiation retinopathy may develop due to Iodine-131, which provides only the results of the fundoscopic examination. 22

For this reason, we aimed to investigate whether RAI treatment affects the posterior segment of the eye.

Ocular complications, such as cataracts, vitreous hemorrhage, and persistent retinal detachment, were observed frequently, particularly in the early period after IBT of large uveal melanomas. These occurrences indicate that limiting radiation damage to the retina and optic nerve remains challenging. 23 24 The primary options to address this issue involve shielding these tissues through plaque design and seed positioning or considering alternative treatment modalities that do not involve irradiation. 25 26

A more posterior tumor location can lead to increased complications such as maculopathy and optic neuropathy due to iodine accumulation in the posterior segment. Conversely, it may also give rise to anterior segment complications like cataracts. This observation aligns with the low radiation tolerance of the lens, which can be exceeded when a thick tumor, even a posterior one, is irradiated with an iodine plaque. The association between tumor height and largest basal diameter with maculopathy and optic neuropathy was found to be weak. 27 28 Moreover, maculopathy and optic neuropathy are partly linked to tumor height and tumor distance from these ocular structures, suggesting that these factors hold potential as dose determinants per macula and optic nerve head. 26

In a study conducted by Kaçar Güveli et al, no retinopathy was observed in any of the patients who received 9,250 to 37,000 MBq Iodine-131 treatment for differentiated thyroid cancer, as assessed through dilated fundus examination of 40 eyes from 20 patients. 22 In our study, unlike this previous study, we evaluated the retina not only with slit-lamp examination but also with OCT, allowing for a more sensitive examination.

Our study revealed no significant side effects related to the accumulation of systemically administered RAI in the retina. We found no significant difference in macular thickness, and RNFLT measured at the beginning and the end of the treatment in both groups.

A limitation of our study is that we did not consider the RAI dose accumulated by the tissues. If the exact accumulation of RAI in the tissues is determined, and a dose–response relationship can be established accordingly, it could significantly contribute to the existing literature.

## Conclusion

This result indicates that RAI therapy, utilized in treating thyroid cancer and hyperthyroidism, does not harm the retina. However, further extensive studies are required to obtain more precise data regarding the potential risk of retinal damage.
